# Ferroptosis as a New Mechanism in Parkinson’s Disease Therapy Using Traditional Chinese Medicine

**DOI:** 10.3389/fphar.2021.659584

**Published:** 2021-06-07

**Authors:** Lijuan Wu, Meijun Liu, Jingtao Liang, Nannan Li, Dongdong Yang, Junjie Cai, Yong Zhang, Yuan He, Zhigang Chen, Tao Ma

**Affiliations:** ^1^ Chengdu University of Traditional Chinese Medicine, Chengdu, China; ^2^ Hospital of Chengdu University of Traditional Chinese Medicine, Chengdu, China; ^3^ Dongfang Hospital, Beijing University of Chinese Medicine, Beijing, China

**Keywords:** Parkinson’s disease, traditional Chinese medicine, iron metabolism, ferroptosis, mechanism of ferroptosis

## Abstract

Parkinson’s disease (PD) is one of the most common neurodegenerative diseases. To date, among medications used to treat PD, only levodopa exhibits a limited disease-modifying effect on early-onset PD, but it cannot delay the progression of the disease. In 2018, for the first time, the World Health Organization included traditional Chinese medicine (TCM) in its influential global medical compendium. The use of TCM in the treatment of PD has a long history. At present, TCM can help treat and prevent PD. Iron metabolism is closely associated with PD. Ferroptosis, which is characterized by the accumulation of lipid peroxides, is a recently discovered form of iron-dependent cell death. The research literature indicates that ferroptosis in dopaminergic neurons is an important pathogenetic mechanism of PD. TCM may thus play unique roles in the treatment of PD and provide new ideas for the treatment of PD by regulating pathways associated with ferroptosis.

## Introduction

Parkinson’s disease (PD) is the second most common neurodegenerative disease and is second only to Alzheimer’s disease. The death toll due to PD increased by 42.4% between 2005 and 2015 (GBD, 2016). According to pathological studies, a reduction in the number of dopaminergic neurons and an abnormal accumulation of α-synuclein (α-syn) result in a shortage of dopamine from the substantia nigra–striatum pathway, causing clinical symptoms, such as tremor, bradykinesia, rigidity, and postural balance disorders (Burbulla et al., 2017; Mittal et al., 2017). The brain is the main tissue in which iron accumulates (Belaidi and Bush, 2016). The development of PD is closely associated with the metabolism and homeostasis of iron in the brain tissue. Ferroptosis is a recently identified iron-dependent cell death pathway that is triggered by a buildup of lipid peroxides and is different from apoptosis (Tiepolt et al., 2018). A lethal buildup of lipid reactive oxygen species (ROS) and an imbalance in oxidation–reduction reactions lead to ferroptotic cell death. Ferroptosis is associated with neurodegenerative disorders, tumors, and cardiovascular diseases. In recent years, ferroptosis has been associated with the pathogenic changes observed in PD.

The goal of the treatment of PD is to relieve symptoms, including motor symptoms and non-motor symptoms, for instance, emotional disorder and pain, by increasing the striatal level of dopamine (Demaagd and philip, 2015). Levodopa is the main treatment for symptoms of PD, although Verschuur et al. (2019) concluded that in patients with early PD treatment with levodopa, it had no disease-modifying effect. However, de Bie et al. (2020) considered that until more effective methods providing stable dopamine concentrations are developed, current evidence supports the use of levodopa as the initial symptomatic treatment in most patients with PD. The use of traditional Chinese medicine (TCM) in the treatment of PD has a long history. In 2018, for the first time, the World Health Organization included TCM in its influential global medical compendium. The treatment of PD using TCM was described in ancient Chinese medicinal texts, for example, in Huangdi Neijing (425–221 BC), the tremor and stiffness are described as “Chan, Chi, Jing.” Later works supplemented this in terms of the preventive treatment and experiences of alleviating the symptoms of PD. In recent years, the results of numerous studies showed that Chinese medicine, Chinese medicine compounds, and single extracts have effects on the regulation of ferroptosis.

## Ferroptosis and Associated Pathways

### Ferroptosis

Ferroptosis is a phenomenon that involves regulated cell necrosis caused by lipid peroxidation induced by iron and ROS (Dixon et al., 2012). The main cellular metabolic mechanisms in ferroptosis include iron metabolism, amino acid metabolism, and lipid metabolism (Friedmann Angeli et al., 2014; Stockwell et al., 2017; Yan and Zhang, 2019). The signaling pathway mediated by glutathione peroxidase 4 (GPx4) (Friedmann Angeli et al., 2014; Yang et al., 2014) and radical-trapping antioxidants (Zilka et al., 2017; Shah et al., 2018) is a classic signal regulation pathway of ferroptosis. Recently, scientists have discovered that the expression of the ferroptosis suppressor protein 1 in cells can significantly protect cells from the adverse effects of factors that induce ferroptosis. Clearly, coenzyme Q10 produced *via* the mevalonate pathway has an antioxidant function in cells, for which it acts as an endogenous inhibitor of ferroptosis by preventing lipid oxidation (Bersuker et al., 2019; Doll et al., 2019) (see Table 1).

**TABLE 1:** Metabolic pathways in ferroptosis.

Metabolic pathway	Main pathway	Metabolite(s)	References
Iron metabolism	Fenton reaction/Haber–Weiss reaction	Fe^2+^	Yan and Zhang (2019); Lei et al. (2019)
Amino acid metabolism	System Xc^−^/GPx4	Glutathione disulphide	Dixon et al. (2012); Friedmann Angeli et al. (2014)
Lipid metabolism	Polyunsaturated fatty acids	Phosphatidylethanolamine-arachidonic acid/adrenic acid	Stockwell et al. (2017); Dixon et al. (2012)
Coenzyme Q10 biosynthesis	Ferroptosis suppressor protein 1/mevalonate pathway	Reduced coenzyme Q10	Doll et al. (2019); Bersuker et al. (2019)

GPx4, glutathione peroxidase 4

### Mechanism of Ferroptotic Cell Metabolism

**FIGURE 1:**
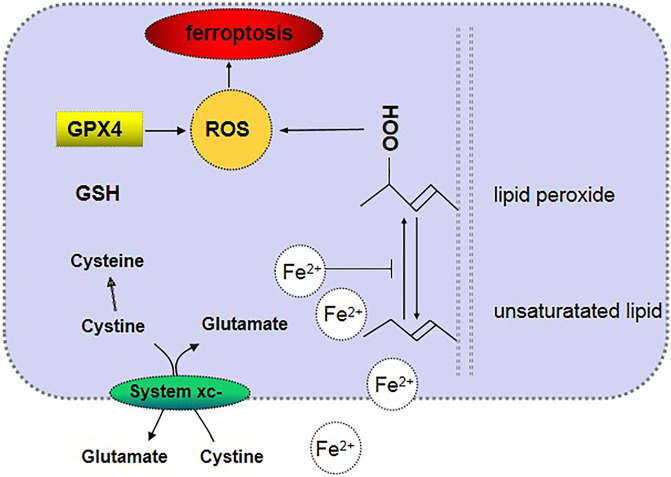
Mechanism of ferroptosis.

Iron metabolism is one of the main mechanisms of ferroptotic cell metabolism. An excess of iron ions in cells can produce lipoxygenase, which causes lipid peroxidation and thus leads to ferroptotic cell death. Excessive amounts of Fe^2+^ in cells generate lipid ROS through the Fenton reaction or Haber–Weiss reaction and thus oxidize cell membrane lipids. Moreover, Fe^2+^ is also an important part of the catalytic subunit of lipoxygenase (Lei et al., 2019). Heme oxygenase-1 is a critical enzyme in heme metabolism and decomposes heme into Fe^2+^ and biliverdin. Heme metabolism, which is an important source of intracellular iron, plays a key role in erastin-induced ferroptotic cell death, such as inducing lipid peroxidation (Kwon et al., 2015). Amino acid metabolism is another crucial metabolic mechanism of ferroptotic cell death because it inhibits the synthesis of glutathione (GSH). System Xc^−^ in the cell membrane transports extracellular cystine and intracellular glutamate in a ratio of 1:1 (Dixon et al., 2012). The intracellular concentration of glutamate is high, and the difference between its concentrations inside and outside the cell is the driving force for its transport. When the neuronal cells in the brain tissue are damaged, system Xc^−^ will be inhibited by a high concentration of extracellular glutamate (Bridges et al., 2012; Friedmann Angeli et al., 2014). Cystine is transported into the cell and then decomposed into cysteine. Then, γ-glutamylcysteine is generated with glutamic acid under the catalytic action of glutamylcysteine ligase. Subsequently, the reaction of γ-glutamylcysteine with glycine is catalyzed by glutathione synthase, which generates GSH (Aoyama and Nakaki, 2015). GPx4 is a key enzyme involved in lipid peroxidation in amino acid metabolism in the metabolic mechanism of ferroptosis, and it can degrade small-molecule peroxides and certain lipid peroxides and inhibit lipid peroxidation (Stockwell et al., 2017). Erastin, which is an inducer of ferroptotic cell death, can hinder the synthesis of GSH by inhibiting the cystine–glutamate exchanger in the plasma membrane and reducing the cellular uptake of cysteine. GSH is necessary for GPx4 lipid repair activity. When GSH is consumed, GPx4 is inactivated, which in turn causes the accumulation of membrane lipid ROS and ferroptosis (Friedmann Angeli and Conrad, 2018). Abnormal lipid metabolism may also lead to ferroptosis (Figure 1). The contents and subcellular locations of amino acids and polyunsaturated fatty acids determine the degree of ferroptosis (Stockwell et al., 2017). Polyunsaturated fatty acids and membrane phospholipids generate arachidonic acid and adrenic acid *via* the esterification of phosphatidylethanolamine (Shindou and Shimizu, 2009), and arachidonic acid and adrenic acid are the main substrates of lipid peroxidation in ferroptotic cell death (Yan and Zhang, 2019).

## Ferroptosis and Parkinson’s Disease

### Iron and Parkinson’s Disease

**FIGURE 2:**
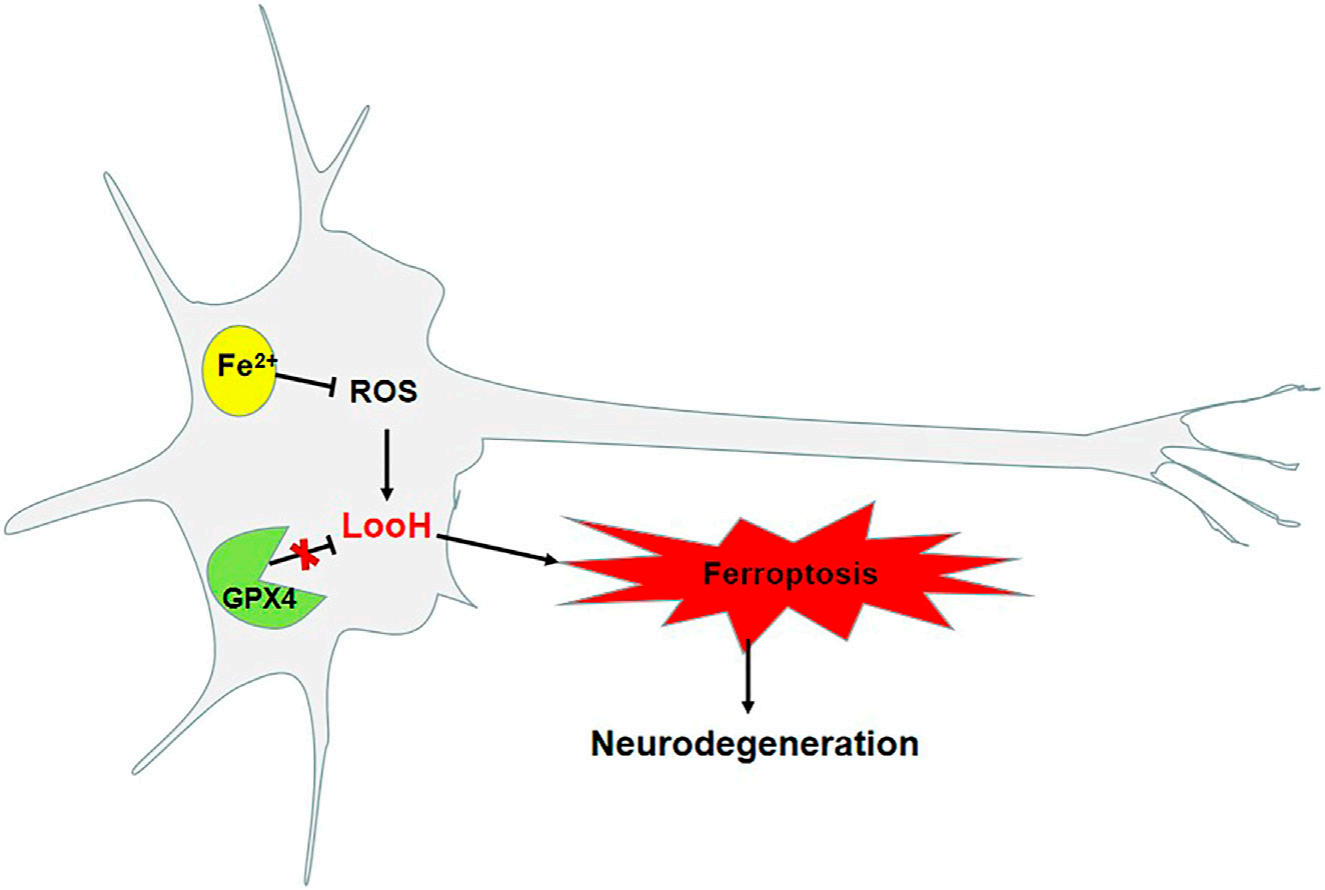
Ferroptosis as a pathway in Parkinson’s disease.

Iron is required for synaptic growth, the formation of myelin sheaths, and the production and transmission activity of neurotransmitters, and is also essential for normal brain development and cognitive functioning (Tiepolt et al., 2018). Iron progressively accumulates in neurodegenerative disorders with age (Abeyawardhane et al., 2018), with greater accumulation observed in the brain, including the nigrostriatal pathway and basal ganglia, namely, the globus pallidus and dopaminergic neurons in the caudate nucleus and putamen. According to pathological studies, a reduction in the number of dopaminergic neurons and an abnormal accumulation of α-syn result in a shortage of dopamine in the substantia nigra–striatum pathway, which causes clinical symptoms, such as tremor, bradykinesia, rigidity, and postural balance disorders. PD is associated with iron, which is particularly abundant in the dopaminergic neurons in the substantia nigra pars compacta (Chmatalova et al., 2017) as an integral component of tyrosine hydroxylase–dependent dopamine synthesis, as well as other enzymatic and nonenzymatic reactions associated with dopamine metabolism (Do Van et al., 2016; Belarbi et al., 2017). Moreover, iron is correlated with an increased risk of the formation of α-syn fibers, which contributes to PD (Ito et al., 2017). The pathology of PD is associated with phospholipid oxidation and changes in iron-regulating mechanisms. Large amounts of Fe^2+^ in organisms are stored in the form of ferritin, whereas the rest of Fe^2+^ is transported through the blood to the cells, which is facilitated by transferrin. The conversion of divalent Fe^2+^ to Fe^3+^ occurs with the reduction of α-syn, and then Fe^3+^ combines with α-syn/C (Duce et al., 2017). The affinity of Fe^2+^ and Fe^3+^ for α-syn is very high, which could accelerate the accumulation of α-syn. The iron chelator, for example, desferrioxamine, can inhibit the abnormal folding of α-syn (Davies et al., 2011). Moreover, ferroportin is the only protein that transports iron into the extracellular space. When iron stored by macromolecules, such as neuromelanin and transferrin, cannot be stored stably, this iron accumulates in cells, and, under its catalytic action, phospholipid hydroperoxidation is induced. This then leads to the damage of dopaminergic neurons, decrease in the production of dopamine, and the disruption of the integrity of the iron metabolism pathway. The accumulation of iron in neurons and glial cells is associated with neurodegenerative disorders (Figure 2). The levels of accumulated iron reflect the clinical status of the disorders (Pyatigorskaya et al., 2015). Astrocytes are essential supporting cells in the neurovascular unit and serve to release iron supplied by the brain capillary endothelial cells to neurons while mitigating its toxicity (Burkhart et al., 2016). To this end, astrocytes are specialized for the export of iron.

### Ferroptosis and Parkinson’s Disease

Ferroptosis is a recently discovered pathogenetic mechanism of PD. Studies have found an increase in iron deposits (Lei et al., 2012; Do Van et al., 2016; Guiney et al., 2017), depletion of GSH (Sian et al., 1994), and lipid peroxidation (Dexter et al., 1989) in the substantia nigra pars compacta. Studies have also found that ferroptosis is involved in the development of MPTP-induced PD in mouse models. Moreover, the administration of the ferroptosis inhibitor ferrostatin-1 can reduce the damage to dopaminergic neurons in models of MPTP- and rotenone-induced PD (Do Van et al., 2016). In neurofibroma cells treated with MPP+, it was found that the inhibition of transferrin receptor 1 can reduce the aggregation of α-syn. Transferrin receptor 1 is an important upregulated protein in ferroptotic cells (Kalivendi et al., 2004). In patients with early PD, treatment with deferiprone can reduce oxidative stress and increase dopamine activity to slow the progression of motor deficits and alleviate existing motor symptoms (Abeyawardhane et al., 2018). In a mouse model of MPTP-induced PD and a model of rotenone-induced cell injury, the administration of the ferroptosis inhibitor ferrostatin-1 can reduce the occurrence of oxidative stress, the deposition of excessive ROS, and the accumulation of α-syn in the substantia nigra and striatum. However, the administration of the ferroptosis inducer erastin can induce the occurrence of ferroptotic cell death, increase the abnormal accumulation of α-syn in mice, and decrease the number of dopaminergic neurons (Friedmann Angeli et al., 2014; Ito et al., 2017).

## TCM in Treatment of Parkinson’s Disease

At present, only a small proportion of the drugs that have a therapeutic effect on PD, such as levodopa, can treat patients with early PD, but these cannot delay the progression of the disease (de Bie et al., 2020). TCM has a long history. PD is classified as “tremor syndrome” in TCM, and Parkinson’s disease–like symptoms have been described at length in ancient Chinese medicine books. PD was described as a disease with the tremor and stiffness in the first medical monograph, namely, *Huangdi Neijing*, more than 2,000 years ago. TCM has obvious curative effects in both the prevention and treatment of diseases by methods, such as formulas (Table 2), acupuncture, and exercises. The Eastern Han dynasty text *Shanghan Zabing Lun* proposed the use of the Zhenwu decoction and Gegen decoction for treating tremors. Later on, physicians have proposed the use of Fangfeng Tongsheng San (1115–1368 AD) and Ding Zhen Wan (1368–1644 AD) for treating Parkinson-related symptoms. At present, many treatment methods continue to be used in later generations and play a beneficial role in the prevention and treatment of PD. In *Huangdi Neijing*, the idea of disease prevention was emphasized. This view is consistent with the early diagnosis and treatment of the prodromal stage of PD, which is currently investigated by medical scientists. The non-motor symptoms of PD, such as rapid eye movement sleep behavior disorder, hypotension, depression, hyposmia, and constipation, appear 5–20 years earlier than the motor symptoms. Preventive intervention in PD during the prodromal period before motor symptoms develop may effectively delay or even reverse the development of the disease. TCM has obvious advantages in the prevention of diseases through overall adjustment.

**TABLE 2:** Classic traditional Chinese medicines with potential benefits for treating Parkinson’s disease–associated symptoms.

Period	Text	Prescription	Decoction composition	Symptom(s)
Han dynasty (206 BC–220 AD)	Shanghan Zabing Lun	Zhenwu decoction	*Smilax glabra* Roxb. (Smilacaceae; Smilax rhizome), *Paeonia lactiflora* Pall. (Paeoniaceae; Paeonia root), *Zingiber zerumbet* (L.) Roscoe ex Sm. (Zingiberaceae; Zingiber rhizomes et root), *Aconitum carmichaelii* Debeaux (Ranunculaceae; Aconitum root tuber), *Atractylodes macrocephala* Koidz. (Asteraceae; Atractylodes rhizome)	Tremor
		Gegen decoction	*Pueraria montana* var. lobata (Willd.) Maesen and S.M.Almeida ex Sanjappa and Predeep (Fabaceae; Pueraria radix et root), *Ephedra sinica* Stapf (Ephedraceae; Ephedra root and rhizome), *Cinnamomum verum* J.Presl (Lauraceae; Cinnamomum terminal branchlet), *Paeonia lactiflora* Pall. (Paeoniaceae; Paeonia root), *Glycyrrhiza glabra* L. (Fabaceae; Glycyrrhiza radix et rhizome), *Zingiber zerumbet* (L.) Roscoe ex Sm. (Zingiberaceae; Zingiber rhizomes et root)	
	Huatuo Shen Shu	Fengshen decoction	*Saposhnikovia divaricata* (Turcz. ex Ledeb.) Schischk. (Apiaceae; Saposhnikovia rhizome), *Hansenia weberbaueriana* (Fedde ex H.Wolff) Pimenov and Kljuykov (Apiaceae; Hansenia radix et rhizome), *Eleutherococcus senticosus* (Rupr. and Maxim.) Maxim. (Araliaceae; Eleutherococcus radix et rhizome), *Paeonia lactiflora* Pall. (Paeoniaceae; Paeonia Pall root), *Panax ginseng* C.A.Mey. (Araliaceae; Panax radix et rhizome), *Coix lacryma-jobi* var. *ma-yuen* (Rom.Caill.) Stapf (Poaceae; Coix ripe kernel), *Scrophularia ningpoensis* Hemsl. (Scrophulariaceae; Scrophularia root), *Ophiopogon japonicus* (Thunb.) Ker Gawl. (Asparagaceae; Ophiopogon radix), *Rehmannia glutinosa* (Gaertn.) DC. (Orobanchaceae; Rehmannia rhizome and root), *Aucklandia costus* Falc. (Asteraceae; Aucklandia radix), *Pinus pinea* L. (Pinaceae; Pinus seed), magnetitum, *Areca catechu* L. (Arecaceae; Areca nut), *Citrus × aurantium* L. (Rutaceae; Citrus fruit), *Achyranthes bidentata* Blume (Amaranthaceae; Achyranthes radix), *Smilax glabra* Roxb. (Smilacaceae; Smilax rhizome), *Cinnamomum verum J.*Presl (Lauraceae; Cinnamomum terminal branchlet)	Spasm and tremor
Tang dynasty (618–907 AD)	Qianjin Yaofang	Bailian decoction	*Ampelopsis japonica* (Thunb.) Makino (Vitaceae; Ampelopsis root), *Zingiber officinale* Roscoe (Zingiberaceae; Zingiber dried rhizome), *Coix lacryma-jobi* var. *ma-yuen* (Rom.Caill.) Stapf (Poaceae; Coix ripe kernel), *Ziziphus jujuba* Mill. (Rhamnaceae; Ziziphus ripe kernel), *Achyranthes bidentata* Blume (Amaranthaceae; Achyranthes radix), *Cinnamomum verum* J.Presl (Lauraceae; Cinnamomum inner bark devoid of cork), *Paeonia lactiflora* Pall. (Paeoniaceae; Paeonia Pall root), *Plantago asiatica* L. (Plantaginaceae; ripe seed), *Glycyrrhiza glabra* L. (Fabaceae; Glycyrrhiza radix et rhizome), *Aconitum carmichaeli* Debeaux (Ranunculaceae; Aconitum root tuber)	Dystonia
Song dynasty (960–1279 AD)	Taiping Huiming Heji Ju Fang	Shexiang Tianma Wan	*Gastrodia elata* Blume (Orchidaceae; Gastrodia rhizome), *Conioselinum anthriscoides* 'Chuanxiong' (Apiaceae; Conioselinum rhizome), *Saposhnikovia divaricata* (Turcz. ex Ledeb.) Schischk. (Apiaceae; Saposhnikovia rhizome),*Chrysanthemum × morifolium* (Ramat.) Hemsl. (Asteraceae; Chrysanthemum flower), moschus, *Arisaema erubescens* (Wall.) Schott (Araceae; Arisaema tuber)	Tremor and dystonia
	Yangshi Jiacang Fang	Xunluo Wan	*Commiphora myrrha* (T.Nees) Engl. (Burseraceae; Commiphora oleo gum resin), *Boswellia carteri* Birdw. (Burseraceae; Boswellia resin exuding from the bark), Tiger bone, *Chinemys reevesii* (Gray) (Testudinidae; Chinemys carapace), *Angelica sinensis* (Oliv.) Diels (Apiaceae; Angelica radix and rhizome), *Trogopterus xanthipes* Milne-Edwards (Petauristidae; Trogopterus droppings), *Aconitum carmichaeli* Debeaux (Ranunculaceae; Aconitum root), *Gastrodia elata* Blume (Orchidaceae; Gastrodia rhizome), *Buthus martensii* Karsch (Scorpio; Buthus dried), *Arisaema erubescens* (Wall.) Schott (Araceae; Arisaema tuber), *Aconitum carmichaeli* Debeaux (Ranunculaceae; Aconitum rhizome), *Eucommia ulmoides* Oliv. (Eucommiaceae; Eucommia bark), *Pheretima pectinifera* Michaelsen (Lumbricus; Pheretima dried), *Clematis chinensis* Osbeck (Ranunculaceae; Clematis radix and rhizome), *Achyranthes bidentata* Blume (Amaranthaceae; Achyranthes radix), *Dipsacus asper* Wall. ex DC. (Caprifoliaceae; Dipsacus root), *Zaocys dhumnades* Cantor (Colubridae; Zaocys dried), *Cistanche deserticola* Ma (Orobanchaceae; Cistanche stem), Cinnabaris	Spasm and difficulty walking
Jin-Yuan dynasty (1115–1368 AD)	Xuanming Lunfang	Fangfeng Tongsheng decoction	*Saposhnikovia divaricata* (Turcz. ex Ledeb.) Schischk. (Apiaceae; Saposhnikovia rhizome), *Rheum officinale* Baill. (Polygonaceae; Rheum radix et rhizome), *Conioselinum anthriscoides* 'Chuanxiong' (Apiaceae; Conioselinum rhizome), *Angelica sinensis* (Oliv.) Diels (Apiaceae; Angelica radix and rhizome), *Paeonia lactiflora* Pall. (Paeoniaceae; Paeonia root), *Mentha canadensis* L. (Lamiaceae; Mentha aerial part), *Ephedra sinica* Stapf (Ephedraceae; Ephedra root and rhizome), *Forsythia suspensa* (Thunb.) Vahl (Oleaceae; Forsythia fruit), Crystallized sodium sulfate, *Scutellaria baicalensis* Georgi (Lamiaceae; Scutellaria root), *Platycodon grandiflorus* (Jacq.) A.DC. (Campanulaceae; Platycodon root), Talc, *Glycyrrhiza glabra* L. (Fabaceae; Glycyrrhiza radix et rhizome), *Nepeta tenuifolia* Benth. (Lamiaceae; Nepeta aerial part), *Atractylodes macrocephala* Koidz. (Asteraceae; Atractylodes rhizome)	Shaking palsy
Ming dynasty (1368–1644 AD)	Zhengzhi Zhunsheng	Jin Ya wine	Jinya Shi, *Bassia scoparia* (L.) A.J.Scott (Amaranthaceae; Bassia fruit), *Rehmannia glutinosa* (Gaertn.) DC. (Orobanchaceae; Rehmannia rhizome and root), *Lepyrodiclis holosteoides* (C. A. Mey.) Fisch. et Mey. (Caryophyllaceae; Lepyrodiclis root), *Aconitum carmichaeli* Debeaux (Ranunculaceae; Aconitum root tuber), *Saposhnikovia divaricata* (Turcz. ex Ledeb.) Schischk. (Apiaceae; Saposhnikovia rhizome), *Asarum sieboldii* Miq. (Aristolochiaceae; Asarum radix and rhizome), *Lllicium Lanceolatum* A. C. Smith (Winteraceae; Lllicium leaf), *Zanthoxylum bungeanum* Maxim. (Rutaceae; Zanthoxylum fruit), *Hansenia weberbaueriana* (Fedde ex H.Wolff) Pimenov and Kljuykov (Apiaceae; Hansenia radix et rhizome)	DystoniaSpasm and difficulty walking
		Ding Zhen Wan	*Gentiana macrophylla* Pall. (Gentianaceae; Gentiana root), *Saposhnikovia divaricata* (Turcz. ex Ledeb.) Schischk. (Apiaceae; Saposhnikovia rhizome), *Nepeta tenuifolia* Benth. (Lamiaceae; Nepeta aerial part), *Achyranthes bidentata* Blume (Amaranthaceae; Achyranthes radix), *Buthus martensii* Karsch(Scorpio; Buthus dried), *Gastrodia elata* Blume (Orchidaceae; Gastrodia rhizome), *Asarum sieboldii* Miq. (Aristolochiaceae; Asarum radix and rhizome), *Conioselinum anthriscoides* 'Chuanxiong' (Apiaceae; Conioselinum rhizome), *Paeonia lactiflora* Pall. (Paeoniaceae; Paeonia root), *Angelica sinensis* (Oliv.) Diels (Apiaceae; Angelica radix and rhizome), *Rehmannia glutinosa* (Gaertn.) DC. (Orobanchaceae; Rehmannia rhizome and root), *Astragalus mongholicus* Bunge (Fabaceae; Astragalus radix), *Atractylodes macrocephala* Koidz. (Asteraceae; Atractylodes rhizome)	
	Qixiao Liang Fang	Da Huoluo Dan	*Dienagkistrodon acutus* (Viperidae; Dienagkistrodon dried), *Zaocys dhumnades* Cantor (Colubridae; Zaocys dried), *Ephedra sinica* Stapf (Ephedraceae; Ephedra root and rhizome), *Asarum sieboldii* Miq. (Aristolochiaceae; Asarum radix and rhizome), *Buthus martensii* Karsch(Scorpio; Buthus dried), *Anemone raddeana* Regel (Ranunculaceae; Anemone leaf), *Paeonia lactiflora* Pall. (Paeoniaceae; Paeonia root), *Cyrtomium fortunei* J.Sm. (Polypodiaceae; Cyrtomium whole plant), *Saposhnikovia divaricata* (Turcz. ex Ledeb.) Schischk. (Apiaceae; Saposhnikovia rhizome), *Pueraria montana* var. lobata (Willd.) Maesen and S.M.Almeida ex Sanjappa and Predeep (Fabaceae; Pueraria radix et root), *Commiphora myrrha* (T.Nees) Engl. (Burseraceae; Commiphora oleo gum resin), *Boswellia carteri* Birdw. (Burseraceae; Boswellia resin exuding from the bark), *Calamus draco* Willd. (Arecaceae; Calamus fruit), Cinnabaris, Rhinoceros (Rhinocerotidae; Rhinoceros cornu), *Pheretima pectinifera* Michaelsen(Lumbricus; Pheretima drried), *Glycyrrhiza glabra* L. (Fabaceae; Glycyrrhiza radix and rhizome), *Syzygium aromaticum* (L.) Merr. and L.M.Perry (Myrtaceae; Syzygium flower), silkworm larva, moschus, *Dryobalanops aromatica* C.F.Gaertn. (Dipterocarpaceae; Dryobalanops resin), *Cinnamomum subavenium* Miq. (Lauraceae; Cinnamomum bark), *Alpinia hainanensis* K.Schum. (Zingiberaceae; Alpinia seed), *Hansenia weberbaueriana* (Fedde ex H.Wolff) Pimenov and Kljuykov (Apiaceae; Hansenia radix et rhizome), Tiger bone, *Scrophularia ningpoensis* Hemsl. (Scrophulariaceae; Scrophularia root), *Bos taurus domesticus* (Bovidae; cattle calculus), *Achyranthes bidentata* Blume (Amaranthaceae; Achyranthes radix), *Gastrodia elata* Blume (Orchidaceae; Gastrodia rhizome), *Agastache rugosa* (Fisch. and C.A.Mey.) Kuntze (Lamiaceae; Agastache leaf), *Bambusa textilis* McClure (Poaceae; Bambusa dried mass of secretion), *Chinemys reevesii* (Gray) (Testudinidae; Chinemys carapace), *Panax ginseng* C.A.Mey. (Araliaceae; Panax radix et rhizome), *Reynoutria multiflora* (Thunb.) Moldenke (Polygonaceae; Reynoutria radix), *Angelica dahurica* (Hoffm.) Benth. and Hook.f. ex Franch. and Sav. (Apiaceae; Angelica root), *Lindera aggregata* (Sims) Kosterm (Lauraceae; Lindera aggregata rhizome), *Styrax tonkinensis* (Pierre) Craib ex Hart. (Dtyracaceae; Styrax resin), *Citrus × aurantium* L. (Rutaceae; Citrus fruit), *Cyperus rotundus* L. (Cyperaceae; Cyperus rhizome and radix), *Wurfbainia vera* (Blackw.) Skornick. and A.D.Poulsen (Zingiberaceae; Wurfbainia fruit), *Davallia trichomanoides* Blume (Polypodiaceae; Davallia rhizome), *Coptis chinensis* Franch. (Ranunculaceae; Coptis rhizome), *Smilax glabra* Roxb. (Smilacaceae; Smilax rhizome), *Scutellaria baicalensis* Georgi (Lamiaceae; Scutellaria root), *Atractylodes macrocephala* Koidz. (Asteraceae; Atractylodes rhizome), *Rehmannia glutinosa* (Gaertn.) DC. (Orobanchaceae; Rehmannia rhizome and root), *Pinus massoniana* Lamb. (Pinaceae; Pinus essential oil), *Rheum officinale* Baill. (Polygonaceae; Rheum radix et rhizome), *Angelica sinensis* (Oliv.) Diels (Apiaceae; Angelica radix and rhizome), *Aucklandia costus* Falc. (Asteraceae; Aucklandia radix), *Aquilaria sinensis* (Lour.) Spreng. (Thymelaeaceae; Aquilaria resin containing wood)	Spasm and difficulty walking

TCM and Western medicine both have their own advantages in the treatment of PD. Part of researches considered Chinese medicine compounds containing medicine ingredients together with Western medicines were superior to single Western medicines in treating PD (Wu et al., 2018). The combination of TCM and Western medicine in the treatment of PD can increase the release of dopamine and improve sensitivity to dopamine, which will relieve the symptoms in patients (Kim et al., 2019). TCM preparations can effectively alleviate the motor symptoms of PD patients and improve their quality of life. Moreover, they can alleviate nausea and vomiting caused by Madopar, the “end-of-dose phenomenon,” the “on–off phenomenon,” and mental disorders (Shantian, 1997; Chen et al., 2012; Zhang et al., 2016). On-medicinal treatments, such as exercises and acupuncture, are beneficial in treating the motor non-motor symptoms of PD. Studies have found that Tai Chi can effectively improve patients’ flexibility, such as their stride length and pace, increase their endurance, enhance their control of their posture and direction, and prevent falls. In fact, Tai Chi plays a useful role in improving the balance and functional ability of patients with early PD and can relieve patients’ non-motor symptoms, depression, and anxiety (Li et al., 2012; Wang and Zhang, 2013; Guan et al., 2016). Acupuncture has a strong clinical effect on the motor symptoms of PD, such as tremor and muscle stiffness, as well as non-motor symptoms, such as disorders of mood and cognition and autonomic dysfunction (Noh et al., 2017).

TCM prescriptions have significant clinical effects in the treatment of PD. The differentiation of syndromes and treatment starts from the pathogenesis of PD and focuses on replenishing the liver and kidney and combining treatments, such as promoting blood circulation to remove blood stasis, extinguishing wind, relieving spasm, and resolving phlegm and detoxification, which can all relieve the motor symptoms of PD and protect the dopaminergic neurons (Wang et al., 2018; Cai et al., 2019; Zhang et al., 2019; Luan et al., 2020). Studies have found that TCM prescriptions and compounds used for the treatment of PD have antioxidant effects, which can affect mitochondrial function and intracellular antioxidant activity, regulate dopamine metabolism and iron metabolism, and protect neurons.

*Pueraria montana* var. lobata (Willd.) Maesen and S.M.Almeida ex Sanjappa and Predeep (Fabaceae; Pueraria radix) can relieve stiff muscles and promote the curative effect on tremor syndrome. Shanghan Zabing Lun proposed the use of Gegen Tang in treating the tension spots in the nape. Several studies have proven that the Guizhi jia Gegen decoction, which comprises *Pueraria montana* var. lobata (Willd.) Maesen and S.M.Almeida ex Sanjappa and Predeep (Fabaceae; Pueraria radix), is beneficial in the treatment of PD and could improve the movement of patients, ameliorate pain, have a good effect on PD patients with sleep disorders, reduce the dosage levels of l-dopamine, and improve the quality of life particularly of advanced patients (Zhu, 2002; Lian and Luo, 2008; Zheng et al., 2018). Its manifold actives intervene in neurological diseases. Puerarin is one of the major medicinal effective ingredients in *Pueraria montana* var. lobata (Willd.) Maesen and S.M.Almeida ex Sanjappa and Predeep (Fabaceae; Pueraria radix). Puerarin may protect dopaminergic neurons in various neurotoxin models of PD (Li et al., 2003; Zhang et al., 2014). It has been found to effectively ameliorate the MPTP-induced motor abnormalities in MPTP-lesioned mice and protect primary rat midbrain neurons against MPP+-induced toxicity *via* progesterone receptor signaling. Progesterone receptor modulates neuroprotective and regenerative responses in Parkinson’s disease and related neurological diseases (Zhao et al., 2020). Another active is *Isodon lophanthoides* var. gerardianus, which lowers the risk of cerebrovascular disease, improves cerebral circulation, and induces the impairment of the neurons (Wang et al., 2008; Lai and Li, 2018).

## TCM Intervenes in Ferroptosis

Chinese medicine is a treasure house of precious natural compounds, and many Chinese medicines have antioxidant effects. Moreover, studies have found that some TCMs can regulate ferroptosis (see Table 3), and the compound preparation of Naotai Fang can improve the cognitive functioning of rats with vascular cognitive dysfunction by regulating ferroptosis (Liao et al., 2015; Zeng et al., 2020). On the basis of the network pharmacological analysis of the ferroptotic cell death–associated targets of TCMs and compound preparations, it was found that quercetin, epigallocatechin-3-gallate, apigenin, luteolin, capsaicin, and genistein can act on multiple ferroptosis-associated target proteins. *Ginkgo biloba* L. (Ginkgoaceae; Ginkgo hojas desecadas), *Eriobotrya japonica* (Thunb.) Lindl. (Rosaceae; Eriobotrya leaves), *Phyllanthus emblica* L. (Phyllanthaceae; Phyllanthus dried ripe fruit), *Pueraria montana* var. lobata (Willd.) Maesen and S.M.Almeida ex Sanjappa and Predeep (Fabaceae; Pueraria radix et root), *Styphnolobium japonicum* (L.) Schott (Fabaceae; Styphnolobium flower bud or flower), *Ephedra sinica* Stapf (Ephedraceae; Ephedra root and rhizome), *Ligustrum lucidum* W.T.Aiton (Oleaceae; Ligustrum ripe fruit), and *Hippophae rhamnoides* L. (Elaeagnaceae; Hippophae ripe fruit) contain more targets that may have greater regulatory effects on the mechanisms of ferroptosis (Ou et al., 2019). Specifically, quercetin has been proven to upregulate the expression of hepcidin, hinder the absorption of intestinal iron, reduce the serum levels of iron, and reduce the bioavailability of iron. It is also an effective natural iron chelator and can reduce tissue damage caused by iron overload by maintaining iron levels in a steady state. Quercetin also removes ROS and other oxidizing substances and is a natural inhibitor of ferroptotic cell death (Sangkhae and Nemeth, 2017; Guo et al., 2018; Mazhar et al., 2018; Xiao et al., 2018). Baicalein, which is another natural ferroptosis inhibitor, can protect against damage due to oxidative stress. In comparison with classic inhibitors, such as liproxstatin-1, ferrostatin-1, β-mercaptoethanol, and deferoxamine mesylate, baicalein has more significant activity against ferroptotic cell death (Xie et al., 2016; Jing et al., 2018). Moreover, artemisinin and piperlongumine are inducers of ferroptotic cell death, and research on their antitumor aspects is increasing. They induce ferroptosis by increasing the ROS levels in tumor cells, reducing GSH levels, interfering with iron metabolism, and increasing Fe^2+^ concentrations (Eling et al., 2015; Greenshields et al., 2016). In comparison with the classic ferroptosis inducers, the TCMs and their active ingredients that have been reported to have a regulatory effect on ferroptosis have a greater number of regulatory targets and stable structures. However, further research is needed to sufficiently support the evidence provided by current studies.

**TABLE 3:** Traditional Chinese medicines that regulate diseases *via* ferroptosis.

Traditional Chinese medicine		Category	Target tissue	Pathway	References
Active ingredient	Baicalein	Inhibitor	Pancreatic cancer cells	GPx4/Fenton reaction	Xie et al. (2016); Jing et al. (2018)
	Artemisinin	Derivant	Tumor cells	Oxidation pathway	Eling et al. (2015)
	Puerarin	Inhibitor	Cardiomyocytes	Oxidation pathway	Roh et al. (2014); Chen et al. (2015)
	Piperlongumine	Derivant	Tumor cells	Oxidation pathway	Greenshields et al. (2016)
	Quercetin	Inhibitor	Intestinal cells	Fenton reaction	Sangkhae and Nemeth (2017); Guo et al. (2018); Mazhar et al. (2018); Xiao et al. (2018)
Compound preparation	Naotai Fang	Inhibitor	Neurons	GPx4/oxidation pathway	Liao et al. (2015); Zeng et al. (2020)
	Huangqin Tang	Inhibitor	Colon cells	GPx4	Wu et al. (2021)
Non-medicinal	Moxibustion	Inhibitor	Neurons	GPx4	Lu et al. (2019)

Moxibustion at the Baihui and Sishencong acupoints in rats in the 6-hydroxydopamine model can increase the expression of the ferroptosis-associated proteins GPx4 and ferritin heavy chain 1 in the substantia nigra while increasing the activity of tyrosine hydroxylase to protect neuronal cells, and the mechanism may be related to the regulation of ferroptosis (Lu et al., 2019). Nevertheless, research on the use of TCM is limited, in particular, Chinese medicines and compound preparations, to treat PD through pathways associated with ferroptosis.

In the process of consulting the literature, we were surprised to have found that Puerarin, the main effective chemical component in the TCM *Pueraria montana* var. lobata (Willd.) Maesen and S.M.Almeida ex Sanjappa and Predeep (Fabaceae; Pueraria radix), is a natural ferroptosis inhibitor and can protect the neurons *via* the inhibited production of ROS. A high concentration of intracellular Ca^2+^ results in the differentiation of Y-79 cells to produce cytotoxic injury (Wang et al., 2016). It can reduce ROS levels, regulate iron homeostasis, and inhibit iron overload, thereby inhibiting ferroptosis (Roh et al., 2014; Chen et al., 2015). Alzheimer’s disease is one of the common neurodegenerative brain disorders. Puerarin has been shown to suppress iron overload in the cerebral cortex and improve spatial learning and memory disorders in mice with Alzheimer’s disease, although the underlying mechanism remains unclear (Zhang et al., 2018). TCM preparations have strong clinical effects on both motor symptoms and non-motor symptoms of PD, and their mechanisms of action mainly focus on antioxidant activity, dopamine metabolism, iron metabolism, and so on. However, research on the use of TCM in the treatment of PD through pathways associated with ferroptosis is limited.

## Conclusion

Ferroptosis is closely associated with various neurodegenerative diseases, and the ferroptosis of dopaminergic neurons is an important recently discovered pathogenetic mechanism of PD. The use of an iron chelator is the latest method for the treatment of PD. Slowing the progression of PD may be achieved by blocking iron-dependent death pathways, for example, upregulating or activating GSH to enable cell repair or inhibiting iron-dependent proline hydroxylase. TCM has a long history in the treatment of PD and has significant effects in the early prevention and clinical treatment of PD. However, research on the use of TCM in the treatment of PD through pathways associated with ferroptosis is limited. Moreover, TCM preparations have strong regulatory effects on ferroptosis to reverse the progress of the disease, which is useful in the development of the treatment methods for PD.

Some evidence on the use of TCM in the treatment of PD through pathways associated with ferroptosis may be insufficient because of the lack of systematic reviews. Moreover, at present, data on the relationship among TCM, PD, and ferroptosis are limited. However, more evidence has proven that TCM can treat other diseases by intervening in ferroptosis, such as tumors, angiocardiopathy, and cerebrovascular and other neurological diseases. These indirect pieces of evidence suggest that ferroptosis may be a valuable pathway in the research of Chinese herbal medicine intervention in PD.

*Pueraria montana* var. lobata (Willd.) Maesen and S.M.Almeida ex Sanjappa and Predeep (Fabaceae; Pueraria radix) has been prescribed for the treatment of PD. Clinical studies showed that the Guizhi jia Gegen decoction was based on *Pueraria montana* var. lobata (Willd.) Maesen and S.M.Almeida ex Sanjappa and Predeep (Fabaceae; Pueraria radix) containing medicine ingredients together which had effectiveness on patients with PD. The active formulations of *Pueraria montana* var. lobata (Willd.) Maesen and S.M.Almeida ex Sanjappa and Predeep (Fabaceae; Pueraria radix) could inhibit the death of nerve cells through the inhibition of enzymatic activities being related to the key genetic PD, and it could induce neuron apoptosis. Puerarin, which is a natural ferroptosis inhibitor, can protect against damage due to oxidative stress and restrain ferroptotic cell death. Moreover, *Pueraria montana* var. lobata (Willd.) Maesen and S.M.Almeida ex Sanjappa and Predeep (Fabaceae; Pueraria radix) and its compounds exhibit a protective effect on nerve cells if they intervene in PD by regulating neuronal ferroptosis. Hence, our future studies will be focused on the development of the treatment methods for PD.
